# Thoracolaparoscopic esophagectomy for esophageal cancer with a cervical or abdominal incision to extract specimen

**DOI:** 10.1097/MD.0000000000031131

**Published:** 2022-10-28

**Authors:** Zhi-Hao Hu, Rui-Xin Li, Jing-Tao Wang, Guo-Jun Wang, Xiu-Mei Deng, Tian-Yu Zhu, Bu-Lang Gao, Yun-Fei Zhang

**Affiliations:** a Department of Gastrointestinal Surgery, The First Affiliated Hospital of Zhengzhou University, Zhengzhou, China.

**Keywords:** abdominal incision, cervical incision, complications, esophageal cancer, thoracolaparoscopic esophagectomy

## Abstract

Surgery is the only curative approach for resectable esophageal cancer. This retrospective study was to investigate the immediate effect and operative complications of conventional and modified thoracolaparoscopic esophagectomy with a cervical or abdominal incision to extract specimen for the treatment of patients with esophageal cancer. Eighty-one patients were enrolled, among which 55 patients underwent conventional McKeown thoracolaparoscopic esophagectomy (conventional MTE) and 26 patients underwent modified MTE with a cerivical incision (modified MTE). The clinical, surgical, and postoperative data were analyzed. No significant (*P* > .05) difference was detected in the clinical data between two groups. The surgical procedure was successful in all patients (100%). The surgical time was significantly (*P* = .018) shorter in the conventional MTE group than in the modified MTE group (280 min vs 317 min). However, no significant (*P* > .05) difference was found in blood loss (200 mL vs 180 mL), intensive care unit (ICU) stay (31.3 ± 11.3 vs 25.2 ± 6.4 hours), first flatus after surgery (2.9 ± 1.9 vs 3.3 ± 1.6 days), postoperative hospital stay (12.9 ± 5.6 vs 12.6 ± 3.3 days), total number of lymph nodes dissected (27.9 ± 4.1 vs 26.7 ± 5.7), types of carcinoma, and pathological classification. No significant (*P* > .05) differences were detected in postoperative complications between the two groups. Assessment of postoperative pain using the visual analogue scale (VAS) score showed a significant (*P* < .05) difference in the VAS score at day 2 (4.81 ± 1.70 vs 3.87 ± 1.14) and day 3 (5.10 ± 0.83 vs 4.61 ± 1.12) between the conventional and modified MTE groups. The modified McKeown thoracolaparoscopic esophagectomy with only one cervical incision is more minimally invasive, more cosmetic, and less painful than the conventional approach.

## 1. Introduction

Esophageal cancer is one of the most common malignant tumors, ranking the sixth cause of cancer-related death in the world.^[1–3]^ For resectable esophageal cancer, radical esophagectomy with complete lymphadenectomy is the preferred treatment option, however, despite advances in surgical technique and postoperative management, the prognosis of patients with esophageal cancer is poor, with the five-year overall survival rate ranging between 15% and 34%.^[4]^ Moreover, the incidence of postoperative complications has been reported ranging 45% to 80%.^[5,6]^ The last decade has witnessed a significant improvement in the mortality and morbidity after application of the minimally invasive esophagectomy (MIE) and neoadjuvant treatment strategies, resulting in improved survival and quality of life.^[7–9]^ Nonetheless, the rate of leakage as a postoperative complication still varies from 4.0% to 20.5% in patients receiving intrathoracic anastomosis and from 12.3% to 31.0% in patients receiving cervical anastomosis.^[3,10–12]^ Intrathoracic leakage is more challenging because of the difficulty of adequate drainage and may result in severe infection before effective measure to heal the leakage. Thus, the MIE McKeown approach with cervical anastomosis may be more popular than that with intrathoracic anastomosis. However, the abdominal incision for surgical specimen extraction is unavoidable in this approach and may impose additional trauma and cosmetic issues. An ideal surgical method may be able to balance surgical trauma and optimal benefits by avoiding additional incision. It was thus hypothesized that a single incision in the neck would be sufficient for esophagastral anastomosis and surgical specimen extraction, avoiding an abdominal incision for specimen extraction and improving the cosmetic effect. However, no comparison has been conducted to investigate the effect and complications of the thoracolaparoscopic McKeown esophagectomy with a single incision in the neck for gastro-esophageal anastomosis and specimen extraction. This study was consequently performed to compare the effect and complications of thoracolaparoscopic McKeown esophagectomy with or without abdominal incision.

## 2. Materials and Methods

This retrospective study was approved by the ethics committee of the First Affiliated Hospital of Zhengzhou University, and all patients or their family members had given the signed informed consent to participate. Between March 2019 and March 2021, patients with esophageal cancers who were treated through the thoracolaparoscopic (McKeown) esophagectomy with or without an abdominal incision were enrolled. The inclusion criteria were patients with esophageal cancer confirmed by endoscopic ultrasonography and biopsy, without distant metastasis confirmed by computed tomography imaging, magnetic resonance imaging, bone scan or Positron emission tomography-computed tomography, treated with the thoracolaparoscopic esophagectomy with or without an abdominal incision for specimen extraction. The exclusion criteria were patients who had received neoadjuvant therapy.

**Figure 1.**
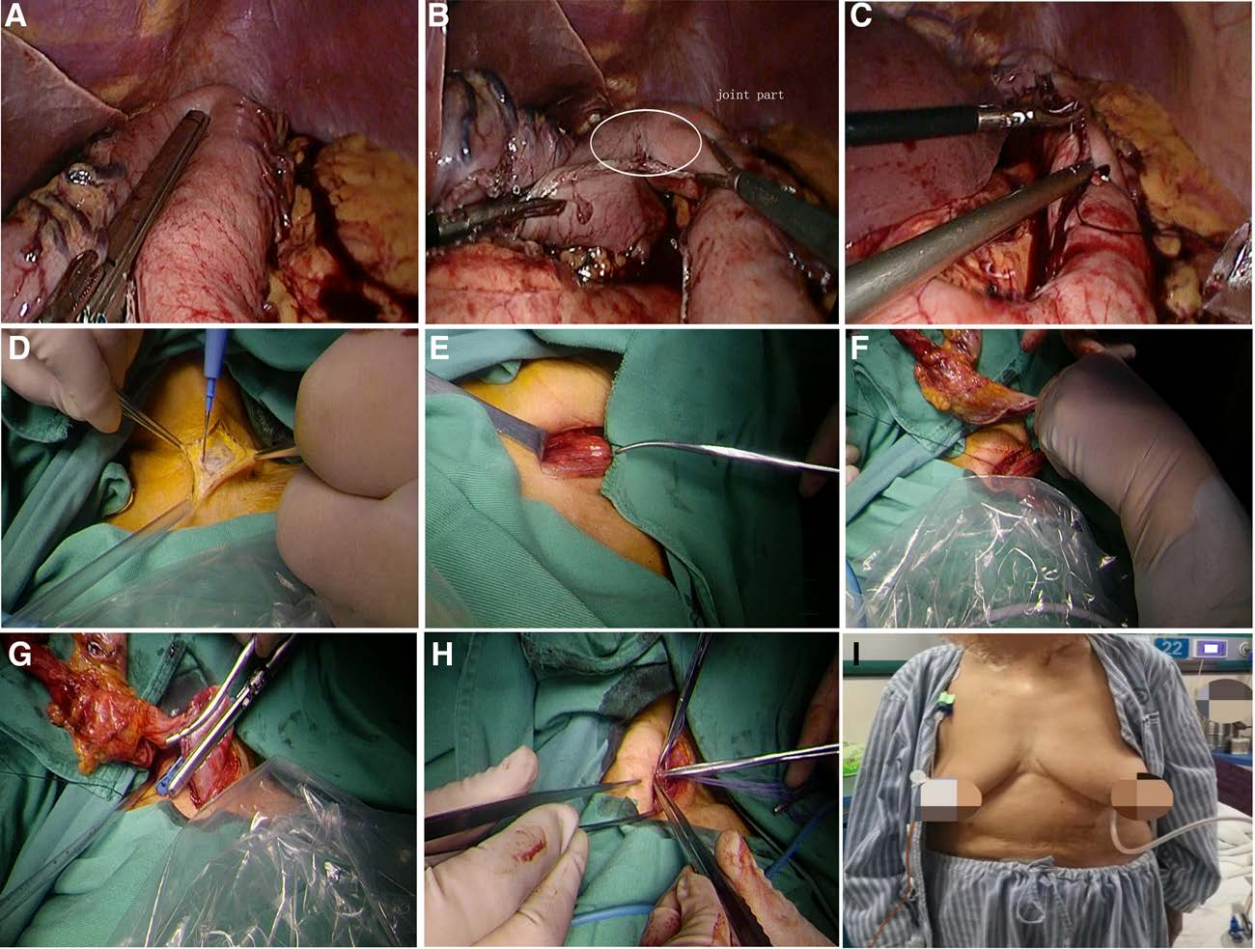
The surgical procedure of modified McKeown thoracolaparoscopic esophagectomy was shown with only one cervical incision. (A) The gastric conduit was constructed. (B) Two parts of the stomach were connected with 3-cm-wide tissues undivided (circle). (C) Laparoscopic reinforcing anastomosis was performed with barbed absorbable suture. (D) A 4 to 5 cm neck incision was made. (E–G) The surgical specimen was removed through the cervical incision. (H) The anastomosis was carried out between the esophagus and the gastric conduit. (I) Postoperative follow-up revealed a linear scar (arrow) on the neck but no surgical scars on the abdominal surface.

The procedure was performed under general anesthesia (Fig. 1). In the conventional McKeown approach, thoracoscopic esophageal mobilization and intrathoracic lymphadenectomy were performed, which was followed by laparoscopic gastric mobilization, construction of gastric conduit, and cervical anastomosis. In this approach, an abdominal incision was made for extraction of surgical specimen before cervical anastomosis. In the modified McKeown approach, no abdominal incision was made, and an oblique incision on the left side of the neck or a transverse cervical incision of 4-5 cm length was made for specimen extraction and gastro-esophageal anastomosis. After construction of the gastric conduit under laparoscopy, the resected stomach remained connected to the gastric conduit at the fundus by 3-cm-wide tissue to facilitate extraction via the cervical incision.

The clinical data, location of esophageal cancer, comorbidities, effect of treatment, and postoperative complications were recorded and analyzed. Postoperative pain was evaluated using the 100 mm visual analogue scale (VAS) one-seven days after surgery.

### 2.1. Statistical analysis

The statistical analysis was performed with the SPSS 20.0 software (IBM, Chicago, IL). Measurement data were presented as mean ± standard deviation if in normal distribution and tested with the Student *t* test or as median and interquartile range if in skew distribution and tested with the Chi square test. Enumeration data were presented as numbers and percentages and tested with the Chi square test. The statistically significant *P* was set at < .05.

## 3. Results

A total of 81 patients were enrolled in this study, among which 55 patients underwent the conventional McKeown thoracolaparoscopic esophagectomy (conventional MTE) and the other 26 patients underwent the modified McKeown thoracolaparoscopic esophagectomy (modified MTE) (Table 1). In the conventional MTE group, there were 37 male and 18 female patients, with a mean age of 64.1 ± 7.6 years, body mass index (BMI) of 25.2 ± 4.3, and the location of cancer in the upper third thoracic segment (n = 3), middle third (n = 40) and lower third (n = 12). In the modified MTE group, there were 18 male and eight female patients, with a mean age of 61.8 ± 9.5 years, BMI 23.41 ± 3.1, and the location of cancer in the upper third thoracic segment (n = 2), middle third (n = 16), and lower third (n = 8). The comorbidities were hypertension in 15, diabetes mellitus in nine, cardiac disease in 15, and pulmonary disease in 12 in the conventional MTE group, whereas hypertension was in five, diabetes in five, cardiac disease in nine, and pulmonary disease in four in the modified MTE. No significant (*P* > .05) difference was detected in the age, sex component, BMI, smoking history, location of cancer, and comorbidities between the two groups.

**Table 1:** Clinical characteristics of the patients in two groups.

Characteristic	CMTE (n = 55)	MMTE-SCI (n = 26)	*P*
Age, yr	64.1 ± 7.6	61.8 ± 9.5	.242
Sex (n, %)			.860
Male	37 (67.3)	18 (69.2)	
Female	18 (32.7)	8 (30.8)	
Body mass index, kg/m^2^	25.2 ± 4.3	23.41 ± 3.1	.075
Smoking history (n, %)	20 (36.4)	11 (42.3)	.568
Location (n, %)			.596
Upper third	3 (5.5)	2 (7.7)	
Middle third	40 (72.7)	16 (61.5)	
Lower third	12 (21.8)	8 (30.8)	
Comorbidity (n, %)			
Hypertension	15 (27.3)	5 (19.2)	.433
Diabetes	9 (16.4)	5 (19.2)	.750
Cardiac disease	15 (27.3)	9 (34.6)	.499
Pulmonary disease	12 (21.8)	4 (15.4)	.497

CMTE = conventional McKeown thoracolaparoscopic esophagectomy, MMTE = modified McKeown thoracolaparoscopic esophagectomy.

Pulmonary disease: different degrees of obstructive ventilation dysfunction.

The procedure was successful in all patients (100%). The surgical time was significantly (*P* = .018) shorter in the conventional MTE group than in the modified MTE group (280 min vs 317 min). However, no significant (*P* > .05) difference was found in blood loss (200 mL vs 180 mL), intensive care unit (ICU) stay (31.3 ± 11.3 vs 25.2 ± 6.4 hours), first flatus after surgery (2.9 ± 1.9 vs 3.3 ± 1.6 days), postoperative hospital stay (12.9 ± 5.6 vs 12.6 ± 3.3 days), total number of lymph nodes dissected (27.9 ± 4.1 vs 26.7 ± 5.7), types of carcinoma, and pathological classification (Table 2).

**Table 2:** Surgical and pathological outcomes (n, %).

Variables	CMTE (n = 55)	MMTE-SCI (n = 26)	*P*
Intraoperative data			
Operation time (min)†	280 (240–326)	317 (292–350)	.018
Blood loss (mL)†	200 (130–220)	180 (142–200)	.158
Postoperative data			
ICU stay (h)*	31.3 ± 11.3	25.2 ± 6.4	.285
First flatus after operation (d)	2.9 ± 1.9	3.3 ± 1.6	.087
Postoperative hospital stay (d)	12.9 ± 5.6	12.6 ± 3.3	.827
Total lymph nodes dissection	27.9 ± 4.1	26.7 ± 5.7	.282
Pathological statistics (n)			.434
pTis	6 (10.9)	5 (19.2)	
pT1aN0	5 (9.1)	3 (11.5)	
pT1aN1	1 (1.8)	0 (0)	
pT1bN0	5 (9.1)	3 (11.5)	
pT1bN1	0 (0)	1 (3.8)	
pT2N0	12 (21.8)	4 (15.4)	
pT2N1	2 (3.6)	2 (7.7)	
pT2N2	2 (3.6)	2 (7.7)	
pT2N3	0 (0)	1 (3.8)	
pT3N0	6 (10.9)	1 (3.8)	
pT3N1	7 (12.7)	2 (7.7)	
pT3N2	6 (10.9)	0 (0)	
pT3N3	2 (3.6)	1 (3.8)	
pT4aN1	0 (0)	1 (3.8)	
pT4aN2	1 (1.8)	0 (0)	
Type of carcinoma(n)			.098
HGD	6 (10.9)	5 (19.2)	
Adenocarcinoma	5 (9.1)	6 (23.1)	
Squamous cell carcinoma	44 (80.0)	15 (57.7)	

CMTE = conventional McKeown thoracolaparoscopic esophagectomy, ICU = intensive care unit, MMTE = modified McKeown thoracolaparoscopic esophagectomy.

† Date in median and interquartile range.

* Data in mean and standard deviation.

The overall complications did not differ significantly between MTE group (35 complications in 28 patients [50.9%]) and modified MTE group (14 complications in 12 patients [46.2%]; *P* > .05). Postoperative complications included pulmonary (16.4% vs 11.5%) and cardiac (7.3% vs 3.8%) complications, anastomotic leakage (14.5% vs 11.5%), recurrent laryngeal nerve injury (5.5% vs 3.8%), gastrointestinal complications (9.1% vs 3.8%), and infection (5.5% vs 3.8%) in the conventional MTE group versus the modified MTE group (Table 3). No significant (*P* > .05) differences were detected in these complications between the two groups. One patient with ileus was surgically managed, and all the other patients with ileus were treated conservatively until complete recovery. Anastomotic stenosis occurred 1 or 2 months after surgery, and 2 patients in the conventional MTE and one in the modified MTE were treated with endoscopic expansion until complete recovery. One patient died of heart failure after surgery, and three patients died of anastomotic leakage and subsequent complications in the conventional MTE group. One patient in the modified MTE group died of anastomotic leakage and subsequent infection. No significant difference existed in the 30-day mortality rate (7.3% vs 3.8%).

**Table 3:** Postoperative complications statistics (n, %).

Variables	CMTE (n = 55)	MMTE-SCI (n = 26)	*P*
Overall complications^a^	28 (50.9)	12 (46.2)	.689
Pulmonary complications	9 (16.4)	4 (11.5)	1.000
Pneumonia	4 (7.3)	3 (11.5)	.830
Pneumothorax	2 (3.6)	0 (0)	1.000
Pleural effusion	5 (9.1)	4 (11.5)	.644
Cardiac complications	4 (7.3)	1 (3.8)	.917
Atrial fibrillation	4 (7.3)	1 (3.8)	.917
Heart failure	1 (1.8)	0 (0)	1.000
Anastomotic leakage	8 (14.5)	3 (11.5)	.983
Recurrent laryngeal nerve injury			
Type I (no therapy)	3 (5.5)	1 (3.8)	1.000
Gastrointestinal complications	5 (9.1)	1 (3.8)	.400
Ileus(conservative)	2 (1.8)	0 (0)	
Ileus (reoperated)	1 (1.8)	0 (0)	
Anastomotic stenosis	2 (3.6)	1 (3.8)	
Infection	3 (5.5)	1 (3.8)	1.000
Wound infection	2 (3.6)	0 (0)	
Central venous catheter infection	1 (1.8)	0 (0)	
Abdominal abscess	0 (0)	1 (3.8)	
30-day mortality	4 (7.3)	1 (3.8)	.917

CMTE = conventional McKeown thoracolaparoscopic esophagectomy, MMTE = modified McKeown thoracolaparoscopic esophagectomy.

a Patients developed complications.

Assessment of postoperative pain using the VAS score showed no significant (*P* > .05) difference in the VAS score at day 1 (3.61 ± 1.33 vs 3.43 ± 1.41) or day 7 (3.12 ± 0.63 vs 2.98 ± 1.16) between the conventional MTE group and the modified MTE group. However, a significant (*P* < .05) difference was detected in the VAS score at day 2 (4.81 ± 1.70 vs 3.87 ± 1.14) and day 3 (5.10 ± 0.83 vs 4.61 ± 1.12) (Table 4).

**Table 4:** Postoperative pains scores on a visual analogue scale (VAS).

Variables	CMTE (n = 55)	MMTE-SCI (n = 26)	*P*
Day 1	3.61 ± 1.33	3.43 ± 1.41	.574
Day 2	4.81 ± 1.70	3.87 ± 1.14	.012
Day 3	5.10 ± 0.83	4.61 ± 1.12	.028
Day 7	3.12 ± 0.63	2.98 ± 1.16	.522

CMTE = conventional McKeown thoracolaparoscopic esophagectomy, MMTE = modified McKeown thoracolaparoscopic esophagectomy.

## 4. Discussion

In this study investigating the safety and immediate effect of conventional and modified MTE with or without abdominal incision for the treatment of patients with esophageal cancer, it was found that the modified MTE with only one cervical incision is more minimally invasive, more cosmetic, and less painful with significantly decreased pain two and three days after the procedure than the conventional approach even though the modified approach may necessitate a longer surgical time.

Surgery is currently the only curative therapeutic approach for resectable esophageal cancer,^[13]^ and the minimal-invasiveness of esophagectomy has been significantly improved by the introduction of MIE, especially thoracolaparoscopic esophagectomy. With MIE, the postoperative complications have also been significantly decreased, including pulmonary infection and poor quality of life, associated with open esophagectomy.^[7,14–16]^ Nonetheless, an abdominal or a thoracic incision has to be created to extract surgical specimen or to construct a gastric conduit, thus adding surgical trauma to the body as well as affecting the cosmetic effect by a big scar on the surface of the body. In the modified MTE in our study, the abdominal incision was avoided by applying one cervical incision only for specimen extraction and gastro-esophageal anastomosis. Moreover, the modified MTE is comparable to the conventional MTE in blood loss, length of stay in the intensive care unit, time of first flatus after surgery, length of postoperative hospital stay, total number of lymph nodes dissected, and frequency and severity of postoperative complications even though the modified MTE necessitated a longer surgical time.

In this cohort, some patients were of Tis or Tia tumors which were still treated with the esophagectomy rather than other less invasive procedures like endoscopic mucosal resection (EMR) or endoscopic submuscular resection (ESMR). Studies have demonstrated that injection of carbon nanoparticles in the submucosa of esophageal upper thoracic segment could label the left gastric artery lymph nodes, which may suggests the lymphatic draining direction from the upper esophagus to left gastric artery lymph nodes in the abdominal cavity.^[17,18]^ This is the theoretical basis to resect the esophageal segment which contains the cancer lesion even in the early stage. We have also treated a patient with an early submucosal carcinoma at the thoracic esophagus, in whom the left gastric lymph nodes were fused into a big mass even though the cancer was still at the early stage. This may suggest the necessity of resecting the upper stomach together with the esophageal cancer itself.^[17,18]^ The use of EMR or ESMR may result in incomplete resection of esophageal submucosal cancers and recurrence later.^[19–29]^ It was demonstrated that in patients with esophageal cancer which was limited only in the submucosal layer, metastases to lymph nodes were more frequent in the upper mediastinum and perigastric area than in the middle or lower mediastinum.^[30]^

Anastomotic complications are the major concern after esophagectomy, including anastomotic leakage and stenosis.^[2,3,10–12]^ In some patients, the overall survival may be closely related to successful anastomosis, avoidance of anastomotic tension, and maintenance of blood supply to the tip of gastric conduit. Great tension and bad blood supply to the gastric conduit tip may lead to a high incidence of anastomotic leakage, resulting in a death risk three times as high as that without leakage and a mortality rate up to 60%.^[2]^ In cervical anastomosis, the gastro-esophageal anastomosis can be performed outside the thorax. In our study, the anastomosis was performed by hand sewing before being pushed back into the thorax. Thus, the anastomosis can be carefully sewed, resulting in few cases of leakage. Nonetheless, no significant difference was found in the frequency of anastomotic leakage or stenosis between the two groups. After investigating factors affecting gastro-esophageal cervical anastomotic leakage, Luo et al found that cervical anastomosis fixation, hypertension, and anastomosis mode are independent risk factors.^[3]^ In their study, cervical gastro-esophageal anastomosis was performed either with hand sewing or with a circular stapler, and the anastomosis was either fixed in the neck by sewing to the adjacent cervical muscles or pushed back in to the thoracic cavity. Pushing the anastomosis back into the thoracic cavity had significantly lowered the incidence of anastomotic leakage compared with the anastomosis being fixed in the cervical muscle (4.4% vs 11.4%, *P* = .027), because pushing the anastomosis back into the thorax could improve both the arterial blood perfusion and venous blood flow return theoretically.^[3]^

The cervical incision was for both gastro-esophageal anastomosis and surgical specimen extraction. After a gastric conduit was made using a linear stapler by dividing the stomach at the lesser curve under laparoscopy, the gastric conduit was pulled into the thoracic cavity through the hiatus, and the gastric specimen was still attached to the stomach fundus by a 3-cm-wide tissue to promote extraction of the specimen via the cervical incision. This process was good for extracting specimen even in patients with a higher BMI.

Using the cervical incision technique, an abdominal incision is avoided, and the cosmetic effect can be improved. Because blood supply is high in the neck, the incision can be healed without forming a large scar. With the emergence and practice of natural orifice specimen extraction surgery, the gastrointestinal surgical procedures have been greatly facilitated, with further improvement of minimal invasiveness as well as improvement of postoperative pain and cosmetic results.^[31–36]^ The modified MTE with one cervical incision only is just like the natural orifice specimen extraction surgery procedure with optimized outcomes like decreased surgical trauma, improvement of postoperative pain and cosmetic effects.

Pain, as the fifth vital sign, is an important clinical symptom and must be evaluated as a fundamental prerequisite to the overall therapeutic effect.^[37,38]^ Pain scales are useful for evaluation of postoperative pain as well as for monitoring treatment effect,^[39,40]^ and most pain evaluations are based on self-reporting of a unidimensional scale representing the subjective pain intensity. The 100 mm VAS and the numerical rating scale are the most commonly used approach for evaluating pain intensity, being relatively easy for both the patient and the evaluator to understand and providing satisfactory sensitivity, reliability, and accuracy.^[41,42]^ In our study, the VAS scoring approach was used to evaluate the pain intensity after MIE for esophageal cancer. The pain was significantly decreased two and three days after the procedure in patients with a cervical incision compared with those with an abdominal incision, indicating more minimal invasiveness. On first day after surgery, no significant (*P* > .05) difference was found in pain between the two groups probably because of application of low-dose analgesics continuously 24 hours after operation.

In recent years, some mediastinoscopic-assisted procedures were also performed with the aim to avoid redundant incisions and decrease surgical trauma,^[43]^ which is consistent with our concept of minimal invasiveness. However, the learning curve of mediastinoscopy is long, and only a few medical centers perform MTE worldwide. Minimally invasive thoracolaparoscopic esophagectomy surgery is still the most widely used mature procedure, and a single cervical incision can be easily made in this procedure for safe extraction of surgical specimen. Mediastinoscopic-assisted esophagectomy can be performed without transthoracic operation or one-lung ventilation, which has minimized the micro- invasiveness of the esophagectomy. The use of one single cervical incision further minimizes the micro-invasiveness of this procedure, suggesting a great advance in achieving good cosmetic effects.

Some limitations existed in this study including one-center and retrospective nature, a small cohort of patients, non-randomization, Chinese patients enrolled only, and no follow-up, which may all affect the generalization of the outcomes. Future studies will have to resolve these issues for improved results.

In conclusion, the modified McKeown thoracolaparoscopic esophagectomy with only one cervical incision is more minimally invasive, more cosmetic, and less painful with significantly decreased pain at days 2 and 3 after the procedure than the conventional approach even though the modified approach may necessitate a longer surgical time.

## Author contributions

**Conceptualization:** Guo-Jun Wang, Yun-Fei Zhang.

**Data curation:** Zhi-Hao Hu, Rui-Xin Li, Jing-Tao Wang, Xiu-Mei Deng, Tian-Yu Zhu, Yun-Fei Zhang.

**Formal analysis:** Zhi-Hao Hu, Rui-Xin Li, Guo-Jun Wang, Xiu-Mei Deng, Tian-Yu Zhu, Bu-Lang Gao.

**Funding acquisition:** Guo-Jun Wang, Yun-Fei Zhang.

**Investigation:** Zhi-Hao Hu, Rui-Xin Li, Jing-Tao Wang, Xiu-Mei Deng, Tian-Yu Zhu, Yun-Fei Zhang.

**Methodology:** Zhi-Hao Hu, Rui-Xin Li, Guo-Jun Wang, Xiu-Mei Deng, Tian-Yu Zhu, Bu-Lang Gao.

**Project administration:** Jing-Tao Wang.

**Supervision:** Xiu-Mei Deng, Bu-Lang Gao.

**Validation:** Zhi-Hao Hu, Rui-Xin Li, Jing-Tao Wang, Guo-Jun Wang, Xiu-Mei Deng, Tian-Yu Zhu, Yun-Fei Zhang.

**Visualization:** Bu-Lang Gao.

**Writing – original draft:** Zhi-Hao Hu.

**Writing – review & editing:** Bu-Lang Gao.
